# Correction to “Modeling the Formation of Degradation Compounds during Thermal Degradation of MEA”

**DOI:** 10.1021/acs.iecr.3c00760

**Published:** 2023-03-17

**Authors:** Lucas Braakhuis, Karen Karolina Høisæter, Hanna K. Knuutila

In the Results section, the reported optimized model parameters in Table 3 are corrected. The model parameters for reaction 2 and reaction 3 should be switched around. This applies to both the reaction rate coefficients at the reference temperature (*k*_ref_) and the activation energy (*E*_A_). The corrected table is shown herein. Since this is only a reporting mistake, the correction does not alter the modeling results or conclusions in the article.

**Table 3:** Optimized Parameters for the Carbamate Polymerization Model

reaction	*k*_ref_ [m^3^·mol^–1^·s^–1^]	*E*_A_ [kJ/mol]
1 (MEA to HEEDA)	1.599 × 10^–11^	151.1
2 (HEEDA to TRIMEA)	1.117 × 10^–10^	121.5
3 (HEEDA to HEIA)	3.054 × 10^–10^	142.6
4 (TRIMEA to AEHEIA)	2.839 × 10^–10^	136.2
5 (MEA to BHEU)	1.281 × 10^–12^	-

